# Local tissue development of type 1 innate lymphoid cells: guided by interferon-gamma

**DOI:** 10.1038/s41392-021-00705-1

**Published:** 2021-07-29

**Authors:** Thomas M. Conlon, Percy A. Knolle, Ali Önder Yildirim

**Affiliations:** 1 Comprehensive Pneumology Center (CPC), Institute of Lung Biology and Disease, Helmholtz Zentrum München, Member of the German Center for Lung Research (DZL), Neuherberg, Germany; 2 Institute of Molecular Immunology and Experimental Oncology, Klinikum rechts der Isar, Technical University of Munich, Munich, Germany

**Keywords:** Innate immune cells, Haematopoiesis

A recent article published in *Science* by Bai et al.^1^ identified interferon-γ (IFN-γ) secreted by type 1 innate lymphoid cells (ILC1s) self-perpetuated their own local development from resident hematopoietic stem cells (HSCs) in adult liver.

**Fig. 1:**
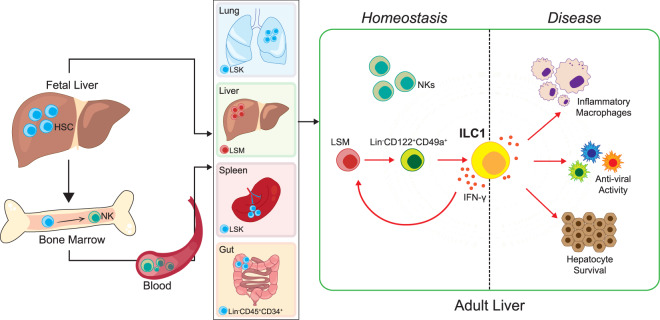
IFN-γ–regulates local development of type 1 innate lymphoid cells (ILC1s) and protects the liver from disease. Lin^−^Sca-1^+^Mac-1^+^ (LSM) hematopoietic stem cells in the adult liver derived from the fetal liver, give rise to Lin^-^CD122^+^CD49a^+^ cells acting as a precursor population whose differentiation potential is restricted to ILC1 cells. Crucially, IFN-γ secreted by ILC1s drives the proliferation and differentiation of IFNγR^+^ LSM progenitor cells to propagate their own development, and contributes to hepatocyte cell survival, anti-viral activity and the generation of inflammatory macrophages. Also depicted are other organs that are sites for extramedullary hematopoiesis (lung, liver, spleen, and gut) along with their HSC phenotype (LSK, Lin^-^Sca1^+^c-Kit^+^)

Hematopoiesis—the development of mature blood cells from multipotent HSCs, changes location throughout life. During embryogenesis, hematopoiesis occurs in the yolk sac before HSCs enter the fetal liver and then seed the bone marrow just before birth. While the bone marrow is the major site of hematopoiesis during adult hood, extramedullary hematopoiesis does occur in other organs like the liver and spleen, but also in lymph nodes, gut, and occasionally lung, where HSCs can reside during adult hood. The liver is a unique organ, in that it contains a large number of tissue resident innate immune cells including γδ T cells, natural killer T cells, conventional natural killer (cNK) cells, type 1, 2, and 3 innate lymphoid cells, which function to maintain liver homeostasis. Here, hepatic ILC1s are cells that appear before birth, are defined as NK1.1^+^NKp46^+^CD49a^+^CD49b^−^ and similar to cNK cells are regulated by the transcription factor T-bet. Unlike cNK cells ILC1s do not express Eomes and lack the cytotoxic capabilities of perforin and granzymes A/B. It was recently demonstrated that ILC1s get activated during acute liver injury and during viral infection of the liver to generate IFN-γ, which subsequently promotes hepatocyte survival^2^ as well as anti-viral activity.^3^ Furthermore, high-fat diet induced metabolic changes promotes group 1 ILC derived IFN-γ, triggering pro-inflammatory macrophage polarization.^4^ However, despite widespread literature on ILC1s showing that they are tissue resident and have tissue-specific functions the source and regulation of tissue resident ILC1s in adult tissue, here in liver, remained an enigma until Bai et al investigated whether it was possible that these cells could develop from local HSCs residing in the liver^1^ (Fig. 1). First, they demonstrated the presence of a population of lineage (Lin)-negative Sca-1^+^Mac-1^+^ (LSM) HSCs in the adult liver of mice similar to what has been described in fetal liver. This population could be reconstituted only by fetal liver cells and not bone marrow cells in chimeric experiments, giving rise to ILC1 cells. Furthermore, experiments with parabiotic mice confirmed LSM cells of adult liver were tissue resident and adoptive transfer of adult liver LSM cells into immunodeficient recipients had the ability to differentiate into multiple lineages but in liver preferentially developed into ILC1s. Delineating further using single-cell RNA-seq analysis coupled with pseudotime trajectory analysis identified a population of liver Lin^-^CD122^+^CD49a^+^ cells downstream of LSM cells acting as a precursor population whose differentiation potential was restricted to ILC1.

To understand the mechanisms underlying extramedullary hematopoiesis, Bai et al.^1^ investigated the involvement of IFN-γ-signaling given its growing importance in regulating cell differentiation and homeostasis, and the observation of reduced liver ILCs in IFN-γ-deficient mice. They confirmed reduced ILCs, particularly ILC1s, in the livers of IFN-γ-deficient mice, while cNK and hepatic lymphocyte numbers remained unchanged. IFNγR1-deficient mice presented the same phenotype, which was restored upon over-expression of IFN-γ. Both liver ILC1s and their Lin^−^CD122^+^CD49a^+^ progenitors were negative for both IFNγR1 and IFNγR2 expression whereas both receptors were heavily expressed on LSM cells. In keeping, IFN-γ-signaling drove the proliferation and differentiation of LSM progenitor cells and not ILC1s themselves, propagating ILC1 generation. Similar to previous studies they also show a clear requirement for T-bet, which was crucial for LSM cell differentiation into ILC1 cells following IFN-γ-signaling. To delineate the source of IFN-γ regulating the development of liver ILC1s they examined CD4, CD8, and B cell deficient animals and found no loss in ILC1 numbers. Finally, conditional knock-out mice lacking IFN-γ in NKp46^+^ ILC cells, which encompasses both cNK and ILC1 cells, suffered from impaired development of liver ILC1s. Crucially, previous work by the group revealed that a deficiency in cNK cells did not affect liver ILC1 number,^5^ thus inferring that IFN-γ secreted by ILC1s in the liver drives their own differentiation from LSM progenitors.

In conclusion, this exciting work by Bai et al.^1^ identified IFN-γ secreted by ILC1s self-perpetuated their own sustained local development from resident HSCs in adult liver that were originally derived from the fetal liver, contributing to our understanding of extramedullary hematopoiesis. These findings lead to an interesting hypothesis that IFN-γ production by ILC1s during acute liver injury, not only promotes hepatocyte survival,^2^ but also leads to the self-perpetuating development of further ILC1s to counteract the ongoing injury. However, these findings raise significant questions that remain to be considered. Firstly, is IFN-γ secreted by ILC1s required for their local development in humans, this is particularly important given the role ILC1s play in regulating liver disease and liver cancers and their potential as novel therapeutic targets. Inline, what is the role of other cytokines like IL-15, which is required for group 1 ILC development and an inducer of IFN-γ. Secondly, T-bet was crucial for LSM cell differentiation into ILC1 cells but a detailed molecular profiling incorporating epigenetic modifications occurring during the differentiation of ILC1s would greatly aid our understanding. Further, ILC1s demonstrate memory responses and contribute to trained immunity, could epigenetic marks during differentiation shape responses later on. Thirdly, does IFN-γ regulate the generation of resident immune cells in various other organs. Namely, IFN-γ levels are increased in many chronic diseases like in the lung during COPD pathogenesis, which are accompanied by increased numbers of circulating ILC1s which correlates with disease severity and exacerbation episodes. One can therefore envisage a “liver-lung axis” where ILC1s generated in the liver from liver resident LSM progenitor cells in response to increased IFN-γ levels contributes to lung pathology. Akin to this, ILC1s are also detected in adipose tissue and the gut highlighting a “gut-liver axis” and/or “adipose tissue-liver axis” as a key regulator in diseases such as obesity and non-alcoholic fatty liver disease. Given the importance of immune cells in the pathogenesis of COVID-19, it will further be interesting to study the role of lung and liver ILC1s also in the context of SARS-CoV-2 infection. The lung epithelium and also liver hepatocytes are targets of SARS-CoV-2 infection and overshooting immune responses can trigger both severe lung and liver damage. ILC1s may contribute to the early defense against viral infection but also to the pathogenesis of immune-mediated tissue damage. In combination with systemic inflammation as a fuel for liver injury in COVID-19 patients, local auto-enhancement of ILC1 generation and cytokine expression may contribute to both liver and lung damage.
